# Epstein–Barr Virus-Positive Mucocutaneous Ulcer: A Unique and Curious Disease Entity

**DOI:** 10.3390/ijms22031053

**Published:** 2021-01-21

**Authors:** Tomoka Ikeda, Yuka Gion, Yoshito Nishimura, Midori Filiz Nishimura, Tadashi Yoshino, Yasuharu Sato

**Affiliations:** 1 Department of Pathology, Okayama University Graduate School of Medicine, Dentistry and Pharmaceutical Sciences, Okayama 700-8558, Japan; me421004@gmail.com (T.I.); p2hq21br@s.okayama-u.ac.jp (M.F.N.); yoshino@md.okayama-u.ac.jp (T.Y.); 2 Division of Pathophysiology, Okayama University Graduate School of Health Sciences, Okayama 700-8558, Japan; gion@okayama-u.ac.jp; 3 Department of General Medicine, Okayama University Hospital, Okayama 700-8558, Japan; nishimura-yoshito@okayama-u.ac.jp

**Keywords:** EBV-positive mucocutaneous ulcer, clinical features, pathological features, immunosuppression

## Abstract

Epstein–Barr virus (EBV)-positive mucocutaneous ulcer (EBVMCU) was first described as a lymphoproliferative disorder in 2010. EBVMCU is a unifocal mucosal or cutaneous ulcer that often occurs after local trauma in patients with immunosuppression; the patients generally have a good prognosis. It is histologically characterized by proliferating EBV-positive atypical B cells accompanied by ulcers. On the basis of conventional pathologic criteria, EBVMCU may be misdiagnosed as EBV-positive diffuse large B-cell lymphoma or other lymphomas. However, its prognosis differs from that of EBV-associated lymphomas, in that patients with EBVMCU frequently show spontaneous regression or complete remission without chemotherapy. Therefore, EBVMCU is now recognized as a low-grade malignancy or a pseudo-malignant lesion. Avoiding unnecessary chemotherapy by distinguishing EBVMCU from other EBV-associated lymphomas will reduce the burden and unnecessary harm on patients. On the basis of these facts, EBVMCU was first described as a new clinicopathological entity by the World Health Organization in 2017. In this review, we discuss the clinicopathological characteristics of previously reported EBVMCU cases, while focusing on up-to-date clinical, pathological, and genetic aspects.

## 1. Introduction

Epstein–Barr virus (EBV) is a member of the herpes virus family, and is one of the most common human viruses [1]. EBV causes latent infection in humans and it may lead to various diseases, including lymphomas and lymphoproliferative disorders (LPDs) even during the latency period. EBV-positive mucocutaneous ulcer (EBVMCU) is a recently established EBV-associated LPD, established in 2010. EBVMCUs are shallow, sharply circumscribed, mucosal, or cutaneous ulcers that are histologically characterized by the proliferation of EBV-positive, variable-sized, atypical B-lymphocytes. This lesion develops in immunocompromised patients, including those who are of advanced age or have iatrogenic immunosuppression, primary immune disorders, or human immunodeficiency virus (HIV)/acquired immunodeficiency syndrome (AIDS)-associated immune deficiencies. EBVMCU has a good prognosis, unlike other EBV-associated LPDs such as EBV-positive diffuse large B-cell lymphoma (DLBCL). Almost all EBVMCU patients achieve remission with the reduction or discontinuation of immunosuppressants, or a watch-and-wait approach. In this review, we discuss the clinical, pathological, and genetic aspects of EBVMCU.

## 2. EBV Biology

EBV, also known as human herpesvirus 4 [2,3,4,5,6], is a ubiquitous virus with a 172 kilobase double-stranded DNA genome [7]. EBV infects both human B lymphocytes and epithelial cells and is transmitted via saliva; therefore, 90% of children get infected with EBV before the age of 5 years. Most people remain asymptomatic, except for those who develop infectious mononucleosis [8,9].

EBV preferentially infects B lymphocytes via engagement between gp350, a viral surface glycoprotein, and its primary receptor on B lymphocytes, that is, complement receptor 2 (CR2/CD21), or CR1 (CD35). gH/gL/gp42, another viral glycoprotein, binds the class II major histocompatibility complex molecules on B lymphocytes, which function as coreceptors. While people may have EBV chronically, it rarely causes clinically significant problems (Table 1).

EBV evades the host immune response by reducing the frequency of viral gene transcription; however, the exact mechanism underlying immune evasion has not yet been elucidated. EBV infection has two phases: a lytic phase and a latent phase. During the lytic phase, the orally transmitted virus replicates in oropharyngeal epithelial cells. The viral genome is then maintained in the host oropharyngeal lymphoid tissue cells during the latent phase. EBV gene products upregulate the expression of various host B cell genes. EBV produces EBV nuclear antigen (EBNA) and latent membrane protein (LMP) gene products during latency. These include EBNA1, EBNA2, EBNA3A, EBNA3B, EBNA3C, EBNA leader protein, LMP1, and LMP2, which mediate the transforming role of EBV in B cells [10].

The latency phase has three subphases, which are defined by the viral gene expression pattern: Latency I, II, and III. In Latency I, EBV expresses only EBNA1, which is present in all infected cells. Viral genome maintenance and replication occur during this phase. Latency II is an intermediate period characterized by the expression of various proteins such as LMP1 and LMP2A/2B, and is further divided into Latency IIa and IIb. Latency IIa is a period of transition to Latency III, and is characterized by LMP1, LMP2A, and EBNA1 expression. In Latency IIa, the virus may provide infected cells the ability to avoid cytotoxic T lymphocyte attack through the mimicry of CD40 and B cell receptor signaling by LMP1 and LMP2A. In Latency IIb, which is characterized by the presence of EBNA2 expression and absence of LMP1 expression, infected cells prepare for transition to Latency III [11]. During Latency III, all the EBV gene products are expressed.

## 3. EBV-Associated LPDs

EBV-associated LPDs are associated with the three latency phases explained in the section “EBV biology”. Latency I is associated with Burkitt lymphoma, Latency II with classic Hodgkin lymphoma (CHL) and extranodal NK/T-cell lymphoma, and Latency III with immunodeficiency-associated LPDs in patients in severely immunocompromised conditions such as HIV/AIDS [1,12].

Immunosuppression is considered to be one of the reasons underlying the emergence of EBV-associated LPDs. For example, Oyama et al. reported EBV-positive DLBCLs in elderly Japanese patients (age > 60 years) who did not have predisposing immunodeficiencies [13]. They suggested that EBV-positive DLBCL could be associated with secondary immunosuppression due to aging. Many cases of iatrogenic immunodeficiency-associated LPDs that developed after methotrexate (MTX) treatment have been reported [1,14]. Histological analysis of MTX-associated LPDs often shows EBV-positive cells. Subsequently, EBVMCU was first described as a distinct clinicopathological entity in 2010 as one of the EBV-associated LPDs occurring in immunosuppressed patients [15].

## 4. What Is EBVMCU?

EBVMCUs are unifocal mucosal or cutaneous ulcers that are histologically characterized by proliferating EBV-positive atypical B cells. Because EBVMCUs are localized lesions, lymphadenopathy or involvement of any other organ is usually not noted. EBVMCUs have mainly been reported in the elderly, those with iatrogenic or primary immunodeficiencies [16,17,18], solid organ or bone marrow transplant recipients [19,20,21,22], and individuals with HIV/AIDS [23]. Lesions often occur because of local trauma, including tooth extraction [24]. In general, patients with EBVMCUs exhibit a good prognosis with spontaneous regression or complete remission after acquiring immunocompetence [1].

According to the World Health Organization disease classification, there are four categories of immunodeficiency-associated LPDs: LPDs associated with primary immune disorders, lymphomas associated with HIV infections, post-transplant LPDs, and other iatrogenic immunodeficiency-associated LPDs. EBVMCU does not belong to any of these four categories; it was first established as a new clinicopathologic entity by the World Health Organization in 2017 [24].

## 5. Clinical Findings for EBVMCU

EBVMCUs are characterized by shallow, sharply circumscribed mucosal or cutaneous ulcers that predominantly occur in oropharyngeal areas without associated systemic symptoms and lymphadenopathy. Of note, EBVMCUs often occur in patients with immunodeficiency.

In the EBVMCU case reports and series in an English-language PubMed search for EBVMCU from 2010 to 2020, we found 186 cases and analyzed all of these cases [5,15,16,17,18,19,20,21,22,23,25,26,27,28,29,30,31,32,33,34,35,36,37,38,39,40,41,42,43,44,45,46,47,48,49,50,51,52,53,54,55,56,57,58,59,60,61,62,63,64,65,66,67,68,69]. The median patient age was 71 years (age range, 0.4–101 years; Table 2) with the male:female ratio of 0.82:1. In the case reports in our search range, EBVMCUs had a slight female predominance. Most previous case reports and series noted that EBVMCU patients were more likely to have autoimmune diseases and be on immunosuppressants.

**Figure 1:**
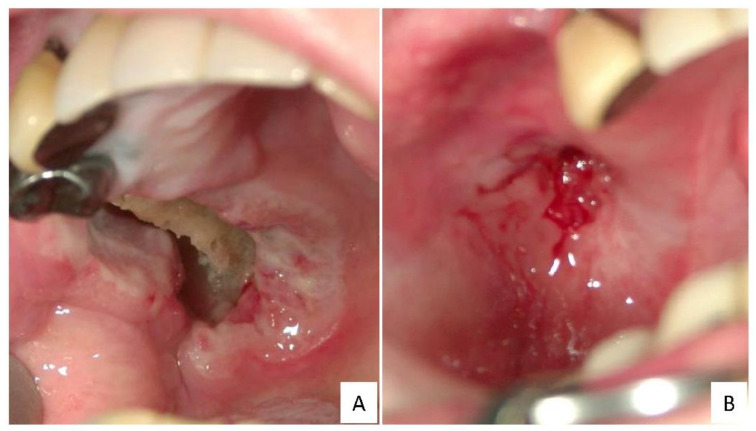
Representative macroscopic findings for EBVMCU in the gingival mucosa. The ulcer developed while the patient was on methotrexate (**A**). After the methotrexate dose was discontinued, the lesion spontaneously disappeared (**B**).

EBVMCUs may emerge when the virus overwhelms the host’s immune response. Ulcer eruption may result from immune abnormalities caused by immunosuppression. For example, in patients who have immunosenescence, iatrogenic immunosuppression or primary immunodeficiencies [16,17,18], or HIV/AIDS [23] or have received solid organ or bone marrow transplants. In these immunosuppressed patients, local trauma, including tooth extraction, could lead to the development of EBVMCUs [24]. Patients exhibit sharply circumscribed mucosal or cutaneous ulcers, with 69.3% of the ulcers occurring in oropharyngeal lesions (Figure 1) [5,15,16,17,18,19,20,21,22,23,25,26,27,28,29,30,31,32,33,34,35,36,37,38,39,40,41,42,43,44,45,46,47,48,49,50,51,52,53,54,55,56,57,58,59,60,61,62,63,64,65,66,67,68,69]. EBVMCUs often develop in the oral cavity and pharynx (including the tonsils), which may be due to the release of EBV into saliva.

The levels of lymphoma prognosis-related laboratory markers, such as soluble interleukin 2 receptor (sIL-2R) and lactate dehydrogenase (LDH) [53], do not increase in patients with EBVMCU. These levels are relatively lower than those in patients with EBV-positive or EBV-negative DLBCL. In a retrospective study, the median LDH value was 212 U/mL (151–397 U/mL) in patients with EBVMCU and 265 U/mL (146–903 U/mL) in patients with EBV-positive DLBCL. The median sIL-2R value was 652 U/mL (263–2786 U/mL) in patients with EBVMCU and 4584 U/mL (460–18,600 U/mL) in patients with EBV-positive DLBCL. In addition, serous sIL-2R levels aid in distinguishing between EBVMCU and systemic EBV-positive LPDs.

In 186 EBVMCU cases [5,15,16,17,18,19,20,21,22,23,25,26,27,28,29,30,31,32,33,34,35,36,37,38,39,40,41,42,43,44,45,46,47,48,49,50,51,52,53,54,55,56,57,58,59,60,61,62,63,64,65,66,67,68,69], during the follow-up periods (1–180 months), spontaneous improvement of the ulcers was noted in eight patients, and 126 patients had either complete or partial remission after discontinuation or dose reduction of immunosuppressants, chemotherapy, or radiotherapy. While eight patients experienced recurrence [15,16,42,53], only four patients had progressive disease [16,18,26,58]. Table 3 summarizes the treatment and outcome of the 186 EBVMCU patients. In view of the frequent spontaneous recovery, patients with EBVMCU are considered to have a favorable prognosis.

## 6. Pathological Findings for EBVMCU

EBVMCUs are characterized by localized mucosal or cutaneous ulcers with EBV-positive atypical lymphoid cells accompanied by dense polymorphic infiltration with various inflammatory cells such as plasma cells, histiocytes, and granulocytes. EBV-positive atypical cells range in size from small to large, and may resemble Hodgkin and Reed–Sternberg (HRS)-like cells. Angioinvasion by these cells is often seen in EBVMCUs. In most cases, EBV-positive cells are positive for CD20 and CD79a and exhibit characteristics of B lymphocytes. These cells are usually also positive for CD30, PAX5, OCT2, and MUM1, with variable expression of BOB1. CD15 is expressed in approximately 50% of cases [1,24].

In some cases of EBVMCUs, the pathological findings are similar to those of DLBCL or CHL. A recent study classified EBVMCUs into four morphological subtypes on the basis of histological features [53].

### 6.1. Polymorphous

**Figure 2:**
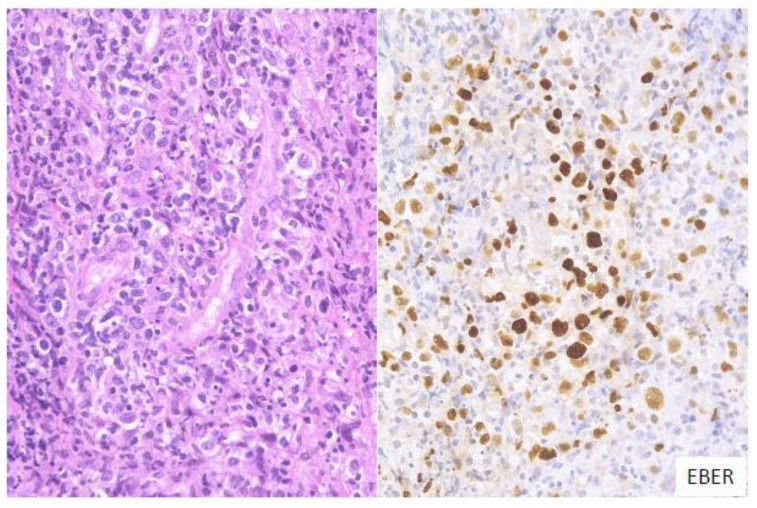
Pathologic findings for polymorphous EBVMCU. A tonsillar EBVMCU in a patient undergoing methotrexate treatment. Atypical lymphoid cells with polymorphous morphology and angioinvasion are seen, and are positive for EBV-encoded small RNA (EBER) (HE and EBER staining; magnification, ×400).

In this subtype of EBVMCU, various small-to-large atypical EBV-positive lymphoid cells, occasionally with a few HRS-like cells, are noted. Atypical lymphoid cells are found in dense clusters or are scattered (Figure 2). Fifty-nine percent of the cases in this study belonged to the polymorphous subtype [53].

### 6.2. Large Cell-Rich

**Figure 3:**
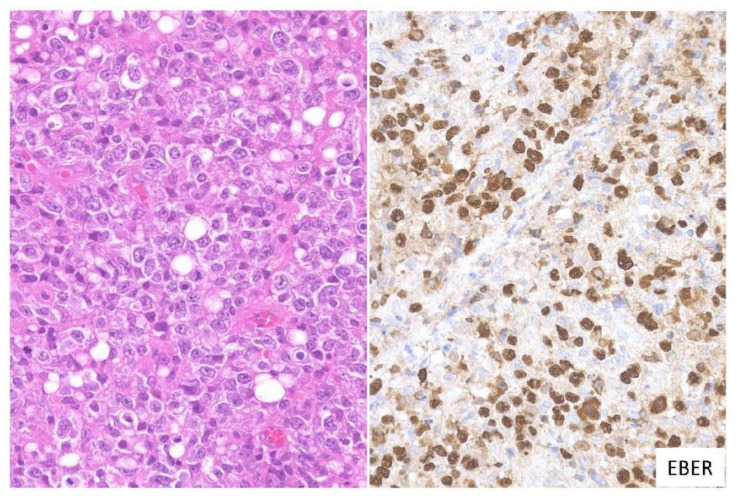
Pathologic findings for large cell-rich EBVMCU. An EBVMCU in the maxilla. The lesion shows monomorphic and dense proliferation of atypical large lymphoid cells resembling diffuse large B-cell lymphoma (DLBCL). In situ hybridization shows atypical cells positive for EBER (HE staining; magnification, ×400).

In this subtype, dense proliferation of large and monomorphic atypical EBV-positive lymphoid cells, similar to that in DLBCL, is noted (Figure 3). Twenty-one percent of the cases in this study belonged to the large cell-rich subtype [53].

### 6.3. CHL-Like

**Figure 4:**
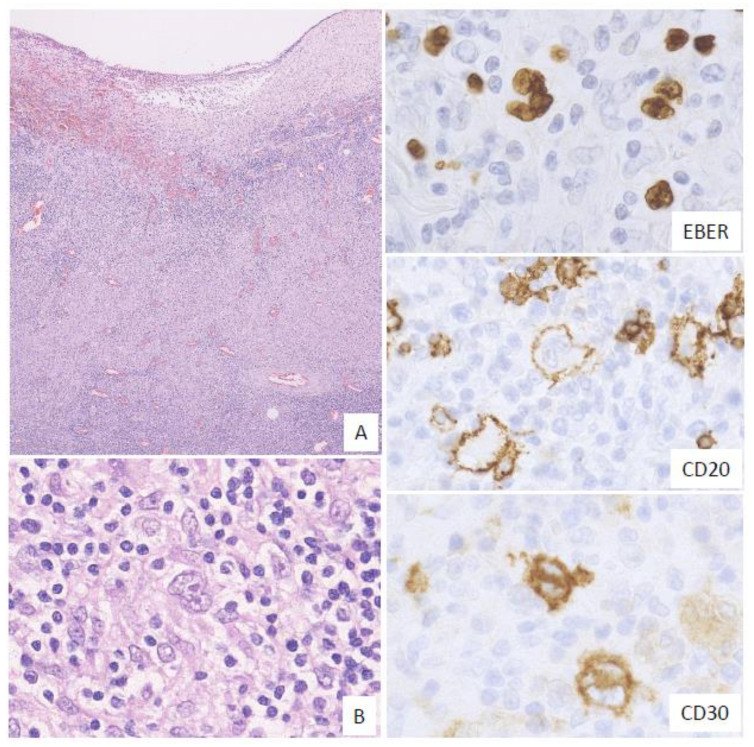
Pathologic findings for classic Hodgkin lymphoma (CHL)-like EBVMCU. A tonsillar EBVMCU in a patient undergoing methotrexate treatment. (**A**) Lymphoid cell infiltration with epithelioid granuloma is observed under the ulcer (magnification, ×40). (**B**) This lesion contains many Hodgkin and Reed–Sternberg (HRS)-like cells (magnification, ×400). The HRS-like cells and other polymorphous atypical lymphoid cells were positive for EBER; the HRS-like cells were also positive for CD20 and CD30 (EBER, CD20, and CD30: magnification, ×400).

In this subtype, many HRS-like EBV-positive cells and various-sized EBV-positive atypical lymphoid cells, sometimes with epithelioid granulomas or eosinophil infiltration, are noted. The HRS-like cells are positive for CD30, similar to the findings for CHL (Figure 4). Twelve percent of the EBVMCU cases were classified as CHL-like [53].

### 6.4. Mucosa-Associated Lymphoid Tissue (MALT) Lymphoma-Like

**Figure 5:**
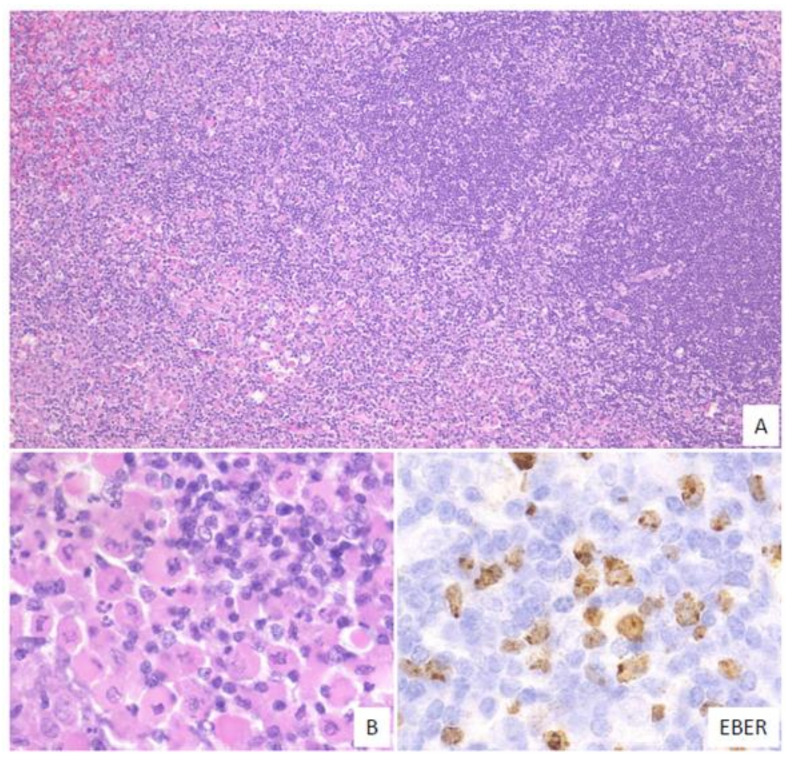
Pathologic findings for mucosa-associated lymphoid tissue (MALT) lymphoma-like EBVMCU. A lingual EBVMCU in a patient undergoing methotrexate treatment. (**A**) The atypical lymphoid cells show centrocytic-like features with plasmacytic differentiation and are proliferating in the expanded interfollicular zone (magnification, ×100). (**B**) Atypical plasmacytic cells with Russell bodies are seen (magnification, ×400). The atypical lymphoid cells are positive for EBER (magnification, ×400).

In this subtype, small-to-medium-sized atypical lymphoid cells that show centrocytic-like features and/or plasmacytic features and proliferate in the expanded interfollicular zone are noted (Figure 5). In our previous study, 9% of the EBVMCU cases belonged to this subtype [53].

Another study reported an EBVMCU case that resembled plasmablastic lymphoma (PBL) [56]. Furthermore, some studies have reported good prognoses among patients diagnosed with HIV-negative PBL [70,71,72,73,74]. Thus, some PBLs, especially HIV-negative cases, may be part of the morphological subtype of EBVMCU. It is challenging to distinguish EBVMCUs from other LPDs, such as EBV-positive DLBCL, solely on the basis of pathological features.

## 7. Genetic Findings for EBVMCU

Genetic studies have been performed to characterize EBVMCUs, including the many reports on detection of clonality in EBVMCUs. In 2010, Dojcinov et al. was the first to report that 38% of the EBVMCUs studied exhibited immunoglobulin heavy chain (IGH) rearrangements [15]. Other studies reported that patients with EBVMCUs had a relatively lower frequency of clonal IGH rearrangements than patients with EBV-positive or EBV-negative DLBCLs [53,75]. In our previous study, clonal IGH rearrangements were detected in 44% of the EBVMCUs, 32% of EBV-positive DLBCLs, and 58% of EBV-negative DLBCLs; these differences were not statistically significant. Thus, IGH rearrangements are not useful for distinguishing between EBVMCU and DLBCL.

Clonal T cell receptor (TCR) rearrangements tend to occur more frequently in EBVMCUs than in EBV-positive and EBV-negative DLBCLs [53]. In our previous study, clonal TCR rearrangements were found in 32% of EBVMCUs, 10% of EBV-positive DLBCLs, and 15% of EBV-negative DLBCLs. Another previous study supported this T-cell clonality; in that study, serum CD8+ T cells increased after MTX reduction in patients who had MTX-LPD and achieved complete remission only due to MTX reduction [76]. Another study showed that B-cell post-transplant LPD was associated with clonal expansion of CD8+ T cells [77]. Thus, we considered that EBVMCU might also be associated with T-cell clonal expansion due to reduced immune surveillance.

Some studies suggested that TCR gene rearrangements could lead to a limited T cell repertoire, which triggers EBV infections in the elderly and in immunocompromised patients [77,78]. The leading players in T cell-mediated immune responses are CD8+ mature memory T cells. In the patient population, T cell epitope recognition might be disabled due to dysfunction of CD8+ mature memory T cells and the CD8+ cells could miss the EBV epitope [78]. This T cell dysfunction might lead to an increase in the number of EBV-positive cells.

Recently, programmed death-ligand 1 (PD-L1) has received attention for its involvement in immune evasion systems. PD-L1 expression in cancer cells suppresses T cell activation, allowing cancer cells to escape the immune surveillance mechanism. Previous studies have shown the presence of PD-L1 expression in most cases of EBV-positive DLBCL [79,80,81]; however, PD-L1 expression was absent in almost all cases of EBVMCUs [64,82]. These results suggest that, in contrast to EBV-positive DLBCLs, EBVMCUs may not possess an immune evasion mechanism.

## 8. Conclusions

EBVMCU is a new disease concept proposed in the last decade. It differs from EBV-associated lymphomas such as EBV-positive DLBCL in that it causes localized lesions and has a good prognosis, although it is challenging to distinguish EBVMCUs from other EBV-positive LPDs solely on the basis of pathological features. Systemic chemotherapy is often unnecessary in patients with EBVMCU. Recognizing the differences between the disease process of EBVMCU and of other associated entities is important for enabling provision of disease-specific treatment and follow-up plans by clinicians and pathologists. Several aspects of the immune mechanism of EBVMCU remain unclear; in particular, the role of T cell clonality and immune checkpoints has not yet been determined. The clinical and pathological features of EBVMCU have been investigated in some studies to date. Therefore, analysis of more EBVMCU cases, with a focus from the side of genetic or molecular aspects, is required in order to elucidate the immune mechanism.

## Figures and Tables

**Table 1:** EBV-associated disorders.

**B-cell Lymphoproliferative Disorders**		
	EBV-positive diffuse large B-cell lymphoma, NOS	
	EBV-positive mucocutaneous ulcer	
	Diffuse large B-cell lymphoma associated with chronic inflammation	
	Lymphomatoid granulomatous	
	Plasmablastic lymphoma	
	Burkitt lymphoma	
	Classic Hodgkin lymphoma	
	Immunodeficiency-associated lymphoproliferative disorders	
		LPD associated with primary immune deficiencies
		Lymphomas associated with HIV
		Post-transplant lymphoproliferative disorders
		Other iatrogenic immunodeficiency-associated lymphoproliferative disorders
**T/NK-cell Lymphoproliferative Disorders**		
	EBV-positive T-cell and NK cell lymphoproliferative diseases of childhood	
	Aggressive NK-cell leukemia	
	Extranodal NK/T-cell lymphoma, nasal type	
	Primary EBV-positive nodal T- or NK-cell lymphoma	
	Chronic active EBV infection	
**Epithelial Cell Malignant Tumors**		
	Carcinoma (nasopharynx, salivary, thymus, lung, stomach, breast, urinary bladder, kidney, uterine cervix, colon)	
**Mesenchymal Tumors**		
	Smooth muscle tumor (leiomyoma/leiomyosarcoma)	
	Inflammatory pseudotumor	
	Inflammatory pseudotumor-like follicular dendritic cell sarcoma	

EBV, Epstein–Barr virus; HIV, human immunodeficiency virus; LPD, lymphoproliferative disorders. Adapted from Ikeda et al. [1] with minor modifications.

**Table 2:** Case reports of EBVMCU (2010–2020) [5,15,16,17,18,19,20,21,22,23,25,26,27,28,29,30,31,32,33,34,35,36,37,38,39,40,41,42,43,44,45,46,47,48,49,50,51,52,53,54,55,56,57,58,59,60,61,62,63,64,65,66,67,68,69].

	Number of Cases (%)	Mean Age (Years)	Age Range (Years)	Sex (Male, %/Female, %)
Iatrogenic Immunodeficiency-Associated EBVMCU (*n* = 119)				
Oropharyngeal	85 (45.7)	73	17–91	32 (37.6)/53 (62.4)
Skin	14 (7.5)	63	47–80	3 (21.4)/11 (78.6)
Gastrointestinal	20 (10.8)	67.5	26–81	14 (70.0)/6 (30.0)
EBVMCU due to age-associated immunosenescence (*n* = 46)				
Oropharyngeal	31 (16.7)	79	51–101	17 (54.8)/14 (45.2)
Skin	4 (2.2)	77	74–88	3 (75.0)/1 (25.0)
Gastrointestinal	11 (5.9)	72	42–84	5 (45.5)/6 (54.5)
EBVMCU with post-solid organ or bone marrow transplant (*n* = 12)				
Oropharyngeal	6 (3.2)	57.5	18–70	4 (66.7)/2 (33.3)
Gastrointestinal	6 (3.2)	57.5	32–70	3 (50.0)/3 (50.0)
HIV/AIDS-Associated EBVMCU (*n* = 2)				
Palate	2 cases (54-year-old male, 36-year-old female)^23^			
Primary Immunodeficiency-Associated EBVMCU (*n* = 4)				
Gingiva	45-year-old female with T-cell deficiency^17^			
Esophagus	61-year-old male with hypogammaglobunemia^18^			
Nasopharyngeal	16-year-old male with CHARGE syndrome^16^			
Skin	5-month-old boy with premature birth^57^			
Chronic Antigenic Stimulation-Associated EBVMCU (*n* = 1)				
Sinus	59-year-old female			
EBVMCU of Unclear Etiology (*n* = 2)				
Oropharyngeal	2 cases (49-year-old female, 49-year-old female) [5,16]			
Total	186	71	0.4–101	84 (45.2)/102 (54.8)

EBVMCU, Epstein–Barr virus-positive mucocutaneous ulcer; HIV/AIDS, human immunodeficiency virus/acquired immunodeficiency syndrome.

**Table 3:** Treatment and clinical courses of 186 EBVMCU cases (2010–2020) [5,15,16,17,18,19,20,21,22,23,25,26,27,28,29,30,31,32,33,34,35,36,37,38,39,40,41,42,43,44,45,46,47,48,49,50,51,52,53,54,55,56,57,58,59,60,61,62,63,64,65,66,67,68,69].

			SR/CR	PR	SD	PD	RR	NR
Iatrogenic Immunodeficiency-Associated EBVMCU (*n* = 119)								
RIS (*n* = 73, 61.3%)			57	10	-	2	-	4
RIS, Rituximab (*n* = 5, 4.2%)			4	-	-	-	1	-
RIS, Chemotherapy (*n* = 6, 5.0%)			6	-	-	-	-	-
RIS, Resection (*n* = 2, 1.7%)			2	-	-	-	-	-
Chemotherapy (*n* = 5, 4.2%)			3	-	-	1	-	1
Antibody therapy (*n* = 1, 0.8%)			0	-	-	-	-	1
Resection (*n* = 8, 6.7%)			1	-	-	-	-	7
None (*n* = 8, 6.7%)			5	-	-	1	2	-
NR (*n* = 11, 9.2%)			2	-	-	-	-	9
EBVMCU due to age-associated immunosenescence (*n* = 46)								
Rituximab (*n* = 1, 2.2%)			1	-	-	-	-	-
Chemotherapy (*n* = 13, 28.3%)			11	-	-	-	-	2
RT (*n* = 4, 8.7%)			4	-	-	-	-	-
Chemotherapy, RT (*n* = 1, 2.2%)			1	-	-	-	-	-
Chemotherapy, RT, and Resection (*n* = 1, 2.2%)			1	-	-	-	-	-
Resection (*n* = 6, 13.0%)			4	-	-	-	-	2
None (*n* = 12, 26.1%)			8	-	1	-	3	-
NR (*n* = 8, 17.4%)			-	-	-	-	-	8
EBVMCU with post-solid organ or bone marrow transplant (*n* = 12)								
RIS (*n* = 6, 50.0%)			6	-	-	-	-	-
RIS, Rituximab (*n* = 3, 25.0%)			3	-	-	-	-	-
RIS, Resection (*n* = 1, 8.3%)			1	-	-	-	-	-
None (*n* = 1, 8.3%)			1	-	-	-	-	-
NR (*n* = 1, 8.3%)			-	-	-	-	-	1
HIV/AIDS-Associated EBVMCU (*n* = 2)								
NR (*n* = 2, 100%)			-	-	-	-	-	2
Primary Immunodeficiency-Associated EBVMCU (*n* = 4)								
Rituximab (*n* = 1, 25.0%)			1	-	-	-	-	-
Rituximab, brentuximab, IVIG (*n* = 1, 25.0%)			-	-	-	1	-	-
Chemotherapy and HSCT (*n* = 1, 25.0%)			-	-	-	-	1	-
Resection (*n* = 1, 25.0%)			1	-	-	-	-	-
Chronic Antigenic Stimulation-Associated EBVMCU (*n* = 1)								
RT (*n* = 1, 100.0%)			1	-	-	-	-	-
EBVMCU of Unclear Etiology (*n* = 2)								
RT (*n* = 1, 50.0%)			-	-	-	-	1	-
Rituximab, RT (*n* = 1, 50.0%)			1	-	-	-	-	-
Total			125	10	1	5	8	37

CR, complete remission; HSCT, hematopoietic stem cell transplant; IVIG, intravenous immunoglobulin; NR, not reported; PD, progressive disease; PR, partial remission; RIS, reduced or discontinued immunosuppressant; RR, relapsing remitting; RT, radiation; SD, stable disease; SR, spontaneous regression, -, no applicable case.

## Data Availability

No new data were created or analyzed in this study.
